# miRNA‐182/Deptor/mTOR axis regulates autophagy to reduce intestinal ischaemia/reperfusion injury

**DOI:** 10.1111/jcmm.15420

**Published:** 2020-06-08

**Authors:** Yunsheng Li, Yanhua Luo, Baochuan Li, Lijun Niu, Jiaxin Liu, Xiaoyun Duan

**Affiliations:** ^1^ Department of Anesthesiology The First Affiliated Hospital of Sun Yat‐sen University Guangzhou China; ^2^ Department of Anesthesiology Zhongshan Ophthalmic Center of Sun Yat‐sen University Guangzhou China

**Keywords:** autophagy, Deptor, ischaemia reperfusion injury, miR‐182, mTOR

## Abstract

It had been reported miR‐182 was down‐regulated after intestinal ischaemia/reperfusion (I/R) damage. However, its role and potential mechanisms are still unknown. This study was aimed to elucidate the function of miR‐182 in intestinal I/R injury and the underlying mechanisms. The model of intestinal injury was constructed in wild‐type and Deptor knockout (KO) mice. Haematoxylin‐eosin staining, Chiu's score and diamine oxidase were utilized to detect intestinal damage. RT‐qPCR assay was used to detected miR‐182 expression. Electronic microscopy was used to detect autophagosome. Western blot was applied to detect the expression of Deptor, S6/pS6, LC3‐II/LC3‐I and p62. Dual‐luciferase reporter assay was used to verify the relationship between miR‐182 and Deptor. The results showed miR‐182 was down‐regulated following intestinal I/R. Up‐regulation of miR‐182 reduced intestinal damage, autophagy, Deptor expression and enhanced mTOR activity following intestinal I/R. Moreover, suppression of autophagy reduced intestinal damage and inhibition of mTOR by rapamycin aggravated intestinal damage following intestinal I/R. Besides, damage of intestine was reduced and mTOR activity was enhanced in Deptor KO mice. In addition, Deptor was the target gene of miR‐182 and was indispensable for the protection of miR‐182 on intestine under I/R condition. Together, our research implicated up‐regulation of miR‐182 inhibited autophagy to alleviate intestinal I/R injury via mTOR by targeting Deptor.

## 1 INTRODUCTION

Intestinal I/R injury is one of the key factors devoting to high mortality in critical patients. It is common in a large number of clinical settings, for example acute mesenteric ischaemia, small intestinal volvulus, small bowel transplantation, shock and trauma.^1^ Intestinal mucosa epithelial cell injury is the key cause of intestinal mucosal barrier dysfunction following I/R, which not only leads to the intestine injury but also causes damage to distal organs. However, the mechanisms of intestine damage induced by I/R have not been entirely elucidated at the molecular level.^2^

In recent, a new type of cell death caused by autophagy, called ‘autophagic cell death’, may contribute to I/R‐induced cell damage. Previous evidence suggested that autophagy was a protective intracellular process. However, current studies have shown that autophagy may contribute benefit or harm in the pathological process of I/R injury.^3^, ^4^ Our previous studies^5^ showed that repression of autophagy reduced intestinal I/R damage and its underlying molecular pathway was associated with mTOR. However, the mechanisms of mTOR regulation in intestinal damage induced by I/R remain unclear.

As a transcription factor, microRNA (miRNA) is functionally important because of its ability of negatively regulating the expression of genes by binding to its target gene with perfect or imperfect complement.^6^ A growing number of studies have shown that miRNA regulation contributes to tissues and organs repair following several events, such as I/R injury.^7^ Our previous research showed miR‐182 was obviously down‐regulated in the intestine following I/R injury,^8^ but its role is still unknown. More studies are needed to better comprehend its function in intestinal damage induced by I/R. Through prediction of target gene, we find Deptor, the natural inhibitor of mTOR,^9^ is a potential target gene of miR‐182. However, it is not yet clear whether miR‐182 is participated in regulating autophagy in intestinal injury through mTOR by targeting Deptor.

Accordingly, the aim of this present research was to elucidate the function of miR‐182 in intestinal I/R damage and the underlying mechanisms. Our hypothesis was that up‐regulation of miR‐182 inhibited autophagy to alleviate intestinal I/R injury via mTOR by targeting Deptor. Our research implicated miR‐182 as a probable curative goal for I/R intestinal disease and provided novel insight into miRNA‐based therapy.

## 2 MATERIALS AND METHODS

### 2.1 Animal care

All mice were used in the light of National Institutes of Health guidelines and were ratified by the Animal Care Committee of Sun Yat‐sen University. The Deptor KO mice were produced by Cyagen Biosciences Inc according to Caron et al study.^10^ All mice were killed with excessive pentobarbital (intraperitoneal injection, 200 mg/kg) at the end of experiment.

Pentobarbital (intraperitoneal injection, 30 mg/kg) was used to anesthetize adult healthy C57BL/6 male mice weighing 20‐25 g. All mice were kept in separate cages in temperature‐controlled rooms that alternated 12‐hour light/dark cycles and acclimated for 1 week before the study. Water was free to all mice, but food was clear away 8 hours before the study. The model of intestinal damage was constructed according to our previous study.^8^ In brief, the small intestine was exteriorized by midline laparotomy, and the intestinal I/R injury was established by occluding the superior mesenteric artery (SMA) with a microvessel clip for 60 minutes followed by 120 minutes reperfusion. Ischaemia was recognized by the existence of pulseless or pale colour of the small intestine. The return of pulses and the re‐establishment of the pink colour were assumed to indicate valid reperfusion of the intestine. After the experiment, a section of 10 cm intestine that was 5 cm from the ileocecal valve was cut and divided into some pieces for test.

### 2.2 Histological evaluation of intestinal damage

Using haematoxylin and eosin to stain the small piece of intestine, the criteria of Chiu's score were used to evaluate the level of intestinal damage.^11^ In brief, mucosal damage was graded from 0 to 5 as follows: grade 0, normal mucosal villi; grade 1, development of subepithelial Gruenhagen's space at the apex of the villus, often with capillary congestion; grade 2, extension of the subepithelial space with moderate lifting of the epithelial layer from the lamina propria; grade 3, massive epithelial lifting down the sides of villi, possibly with a few denuded tips; grade 4, denuded villi with lamina propria and dilated capillaries exposed, possibly with increased cellularity of lamina propria; and grade 5, digestion and disintegration of the lamina propria, haemorrhage and ulceration. Two pathologists blinded to the experimental groups evaluated damage of intestinal mucosa independently. To evaluate intestine damage, at least five fields choose at random from each mouse were assessed and averaged.

### 2.3 Evaluation of level of diamine oxidase (DAO)

As a marker sensitized to intestinal mucosal damage, serum DAO was measured at the wavelength of 436 nm with an ultraviolet spectrophotometer by using a chemical analysis kit in line with the manufacturer's instruction.

### 2.4 Administration of agomir‐182 and antagomir‐182

Mice were received agomir‐182 or antagomir‐182 or their NC injections via the tail vein for three consecutive days. RiboBio produced agomir‐182 and antagomiR‐182. Their orders were listed as follows.

agomir‐182:

5′UUUGGCAAUGGUAGAACUCACACCG3′

3′AAACCGUUACCAUCUUGAGUGUGGC5′

agomir‐182 NC:

5′UUUGUACUACACAAAAGUACUG3′

3′AAACAUGAUGUGUUUUCAUGAC5′

antagomiR‐182:5′‐CGGUGUGAGUUCUACCAUUGCCAAA‐3′

antagomiR‐182 NC: 5′‐CAGUACUUUUGUGUAGUACAAA‐3′

### 2.5 Administration of rapamycin and 3‐methyladenine (3‐MA)

Rapamycin, which was dissolved in dimethyl sulphoxide (DMSO), was intraperitoneally injected (2 mg/kg, 1 mL) 30 minutes before ischaemia. 3‐MA dissolved in ddH_2_O was intraperitoneally injected (2 mg/kg, 1 mL) 30 minutes before ischaemia.

### 2.6 Real‐time quantitative polymerase chain reaction

Using Trizol^®^ Reagent to extract RNA from fresh tissue in line with the manufacturer's strategy, the UV–Vis spectrophotometer UV‐1800 was used to examine the quality of RNA. 1.5% agarose gel electrophoresis with OD260/280 between 1.8 and 2.0 and RNA 28s/18s>1 was used to verify the RNA integrity. Following the manufacturer's protocol, using RT primer and TaqMan probe of miRNAs on an ABI 7500 to carry out miRNA analysis, the endogenous reference control was performed by RNA U6 small nuclear 2 expression. Every RT‐qPCR examination was duplicated doubly, using three autonomous samples. The comparative abundance of miRNA or mRNA in intestinal mucosa was counted using the equation RQ = 2^−ΔΔCT^. The primers were as follows: Deptor forward primers 5′‐AGCAGAGAGAGCTGGAACGC‐3′, reverse primers 5′‐CAGAGGCCTCCTTATGTTCA‐3′; and U6 forward primers 5′‐ACTGCCGCATCCTCTTCCT‐3′ and reverse primers 5′‐TCAACGTCACACTTCATGATGGA‐3′; miR‐182 forward primers 5′‐UUUGGCAAUGGUAGAACUCACACCG‐3′, and reverse primers 5′‐AGUGUGAGUUCUACCAUUGCCAAA‐3′.

### 2.7 Ultrastructure detection by electronic microscopy

Intestinal segments were cut into 1 mm cubes rapidly and were soaked in 2.5% glutaraldehyde in 0.1 mol/L phosphate buffer at 4°C overnight and then fixed in 1% buffered osmium tetroxide for 1.5 hours. Samples were compared with 5% uranylacetate for 2 hours, dehydrated by a ranked ethanol series and embedded in epoxyresin. The samples were regularly processed and detected under an electronic microscopy. As the morphological characteristic of autophagy, autophagosome is the endocellular degradation form of cytoplasmic content sequestered by smooth double membranes without ribosomes. The number of autophagosomes was assessed and averaged at least six random selective fields from each mice.^5^

### 2.8 Western blot analysis

In short, specimens were homogenized in extraction buffer that was ice‐cold. After centrifugation, supernatants were collected from the homogenates; proteins were separated by SDS‐PAGE and then transferred onto membrane, finally hatched with adequate primary antibody for LC3‐I/LC3‐II, phodpho‐S6 (pS6), S6, p62, Deptor and β‐actin. Photographic films were used to capture images. The images were scanned, and typical outcomes were presented. By using Image J v1.46r, the optical density of Western blots was quantified by standardized β‐actin strength.

### 2.9 Dual‐luciferase reporter assay

Firstly, 293T cells were cultivated in 48‐well plates. After 24 hours, they were transfected with plasmid which contained PMIR‐RB‐REPORT‐Deptor3‐3′‐UTR (1 μg/well) and miR‐182 mimic (100 nm per well) or their negative control, separately. As the reference gene, the plasmid contained a synthetic firefly luciferase gene. As the reporter gene, the plasmid contained Deptor 3′‐UTR downstream of Renilla luciferase. The cells were gathered 24 hours after transfection, using a dual‐luciferase reporter assay kit to assess the ratio of Renilla and Firefly luciferase activities in line with the manufacturer's instructions.

### 2.10 Statistics

All data were presented as mean ± standard deviation (SD). Student's *t* test was used to analyse differences between two groups. One‐way ANOVA post hoc procedure (Tukey post‐test) was used to analyse differences among multiple groups. Statistical significance was defined as *P* < .05 (two‐sided tests).

## 3 RESULTS

### 3.1 miR‐182 is down‐regulated after intestinal I/R and is regulated by agomir‐182 or antagomir‐182

**FIGURE 1:**
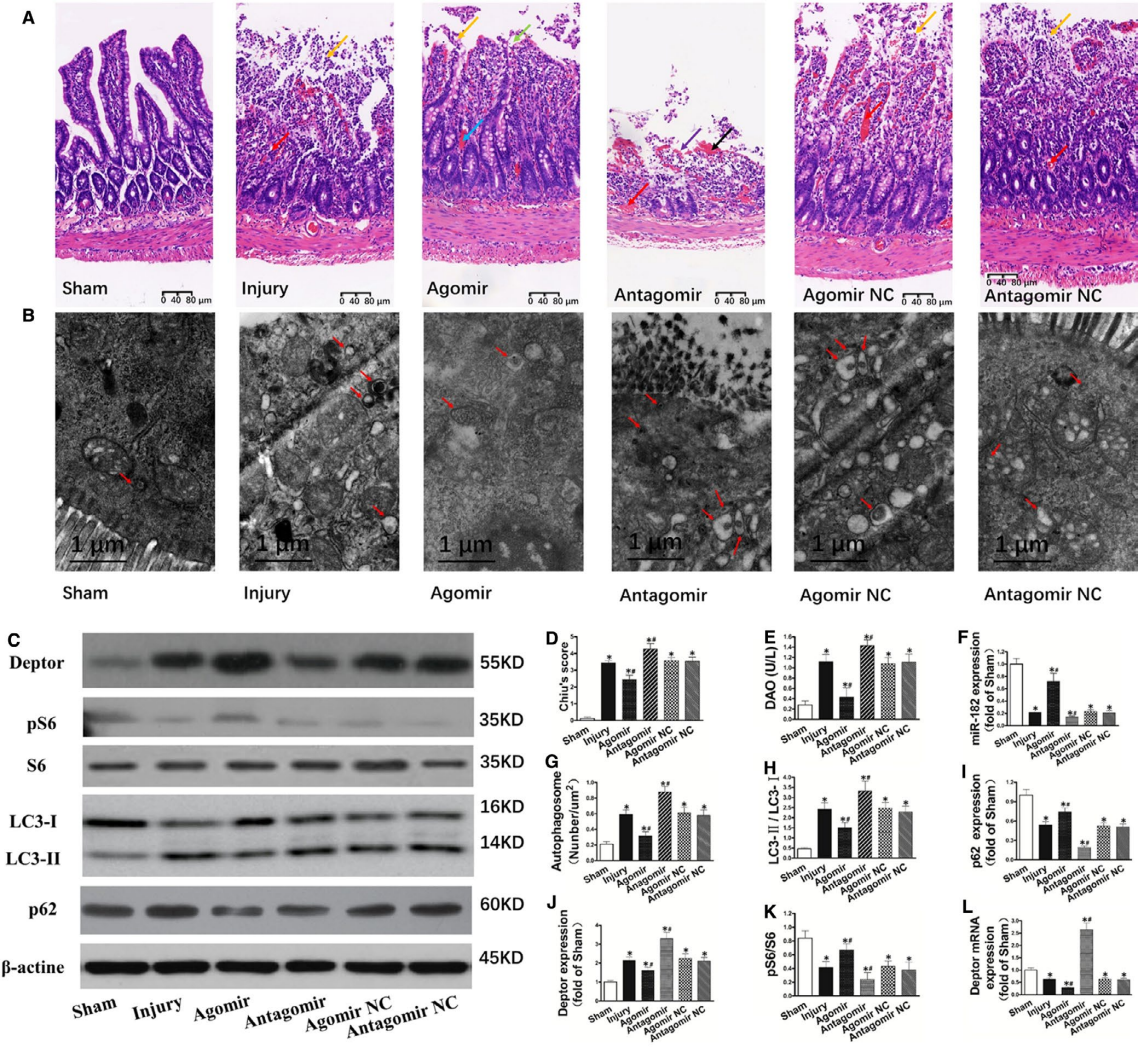
Up‐regulation of miR‐182 reduces intestinal injury, autophagy, Deptor expression and enhances mTOR activity after intestinal I/R. C57BL/6 mice (8‐10 wk old) underwent sham operation or SMA occlusion for 60 min followed by 120 min reperfusion. The mice received injections of agomiR‐182, antagomiR‐182 or theirs NC via the tail vein (40 mg/kg, 100 μL) for three consecutive days. The intestinal I/R injury model was established on the fourth day after injection. A, Histopathologic changes of the intestinal mucosa (haematoxylin and eosin staining). The intestinal mucosa was intact in the Sham group, whereas massive epithelial lifting down the sides of villi, denuded villi with lamina propria and dilated capillaries exposed, possibly with increased cellularity of lamina propria were observed in the Injury, Agomir NC and Antagomir NC group. Development of subepithelial Gruenhagen's space at the apex of the villus, some with capillary congestion, extension of the subepithelial space with moderate lifting of the epithelial layer from the lamina propria and massive epithelial lifting down the sides of villi were observed in the Agomir group. Haemorrhage and ulceration were observed in the Antagomir group. B, Changes of autophagosome under electronic microscopy examination. Autophagosome was pointed by red arrow. C, Representative electrophoresis pattern of Deptor, pS6, S6, LC3‐I, LC3‐II and p62. D‐K, Changes of Chiu's score, DAO, miR‐182 expression, autophagosome, LC3‐II/LC3‐I, p62, Deptor, pS6/S6 and Deptor mRNA, respectively. L, Upregulation of miR‐182 reduces intestinal injury, autophagy, Deptor expression, Deptor mRNA expression and enhances mTOR activity after intestinal I/R. The data were expressed as the mean ± SD (n = 8). *P < .01 compared with the Sham group, ^#^ *P *< .05 compared with the Injury group

Representative intestine sections revealed that expression of miR‐182 was remarkably decreased in intestine mucosa following I/R injury (Figure 1F). To clarify the function of miR‐182 in intestinal damage induced by I/R, we either up‐regulated or down‐regulated expression of miR‐182 in mice, respectively. Compared with Injury group, expression of miR‐182 was markedly up‐regulated when pretreated with agomir‐182 while was markedly down‐regulated when pretreated with antagomir‐182 (Figure 1F).

### 3.2 Up‐regulation of miR‐182 reduces intestinal injury, autophagy, Deptor expression and enhances mTOR activity after intestinal I/R

Histological assessments showed significant intestinal mucosal injury was seen in Injury group (Figure 1A). On the contrary, natural mucosal structure was detected in Sham group. Mild damage was seen when up‐regulation of miR‐182 while more serious injuries were seen when down‐regulation of miR‐182. In line with the histological alteration, Chiu's score and the DAO activity were obviously higher in Injury group than in Sham group. Compared with Injury group, Chiu's score and DAO levels were significantly reduced when up‐regulation of miR‐182 while were further increased when down‐regulation of miR‐182 (Figure 1D,E). These results illustrated that up‐regulation of miR‐182 reduced intestinal damage induced by I/R.

Compared with the Sham group, autophagy in mucosa of intestine was obviously increased after suffered I/R demonstrated by the significantly enhanced LC3‐II/LC3‐I and autophagosomes and reduction in p62 expression. In comparison with Injury group, autophagy was significantly increased when down‐regulation of miR‐182. On the contrary, when up‐regulation of miR‐182, autophagy was significantly decreased compared with Injury group (Figure 1B,C,G,H,I). These results indicated up‐regulation of miR‐182 reduced autophagy following intestinal I/R.

In addition, in comparison with the Sham group, DEP domain‐containing mTOR‐interacting protein (Deptor) and Deptor mRNA expression were significantly decreased. However, activity of mTOR was significantly increased demonstrated by the significantly increased pS6/S6 after suffered I/R. Compared with Injury group, Deptor and Deptor mRNA expression were obviously increased in Agomir group while was significantly decreased in Antagomir group (Figure 1J,L). Compared with Injury group, activity of mTOR in intestine was obviously inhibited in Agomir group while was significantly enhanced in Antagomir group (Figure 1K).

### 3.3 Inhibition of autophagy reduces intestinal damage

**FIGURE 2:**
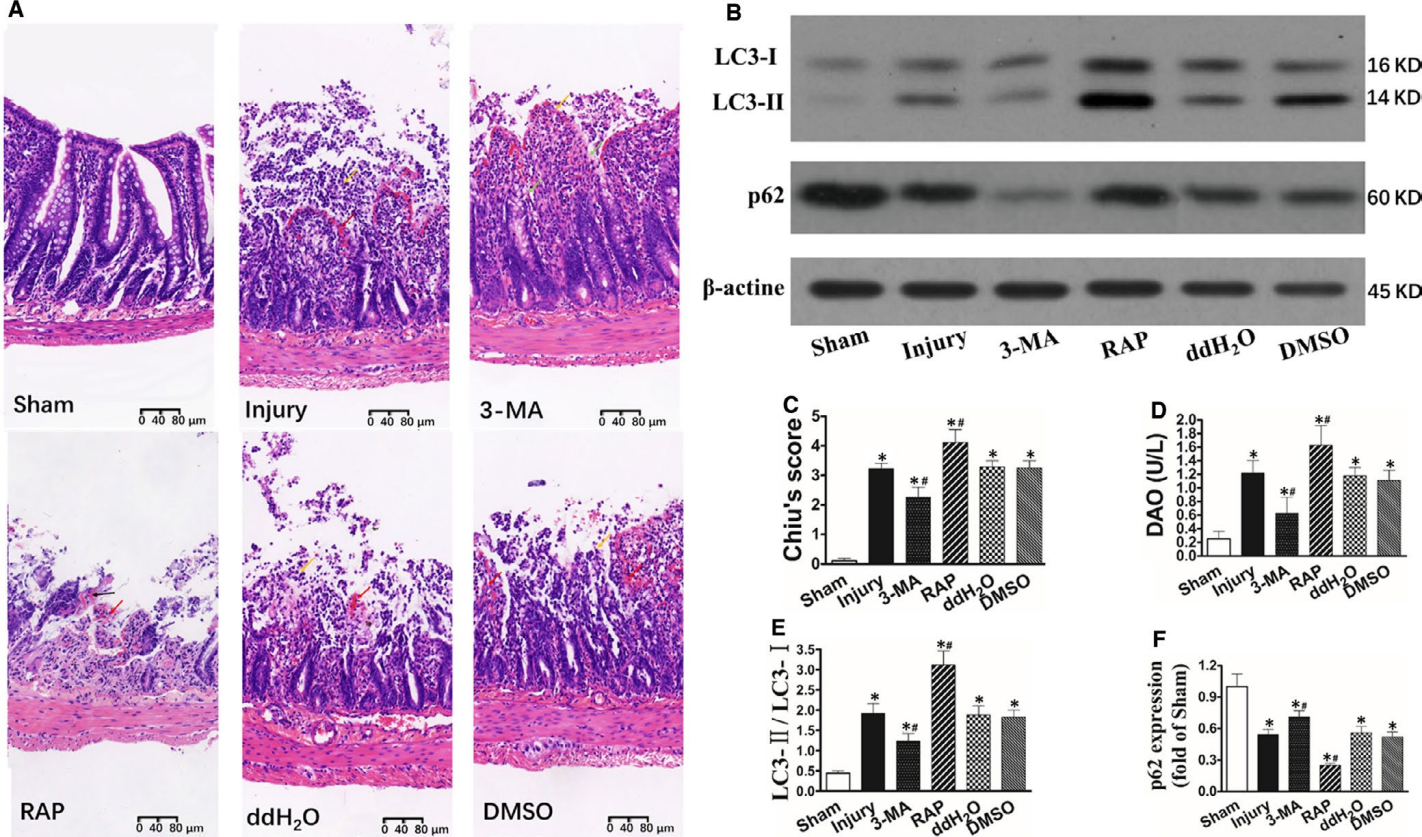
Inhibition of autophagy attenuated intestinal injury after intestinal I/R. C57BL/6 mice (8‐10 wk old) underwent sham operation or SMA occlusion for 60 min followed by 120 min reperfusion. 3‐MA was dissolved in ddH_2_O was intraperitoneally injected (2 mg/kg, 1 mL) 30 min before ischaemia. Rapamycin was dissolved in dimethyl sulphoxide (DMSO) and was intraperitoneally injected (2 mg/kg, 1 mL) 30 min before ischaemia. A, Histopathologic changes of the intestinal mucosa (haematoxylin and eosin staining). The intestinal mucosa was intact in the Sham group. Histopathologic changes in the Injury, ddH_2_O and DMSO group were similar to the Agomir NC group. Histopathologic changes in 3‐MA were similar to the Agomir group. Haemorrhage and ulceration were observed in the RAP group. B, Representative electrophoresis pattern of LC3‐I, LC3‐II and p62. C‐F, Changes of Chiu's score, DAO, LC3‐II/LC3‐I and p62, respectively. The data were expressed as the mean ± SD (n = 8). *P < .01 compared with the Sham group, ^#^ *P *< .05 compared with the Injury group

In order to investigate the function of autophagy in intestinal damage, autophagy inhibitor 3‐MA^11^ was treated prior to ischaemia. Compared with the Injury group, autophagy was obviously declined in 3‐MA group (Figure 2B,E,F). Intestinal damage was reduced demonstrated by decline histological assessments, Chiu's score and DAO level (Figure 2A,C,D). To further verify the function of autophagy in intestinal damage, rapamycin, an autophagy inducer and mTOR inhibitor,^11^ was treated prior to ischaemia. The results showed that autophagy level was increased and intestinal injury was further deteriorated compared with Injury group. Taken together, these results suggested that inhibition of autophagy reduced intestinal damage and inhibition of mTOR aggravated intestinal damage following I/R.

### 3.4 Intestinal damage is reduced, and mTOR activity is enhanced in Deptor KO mice

**FIGURE 3:**
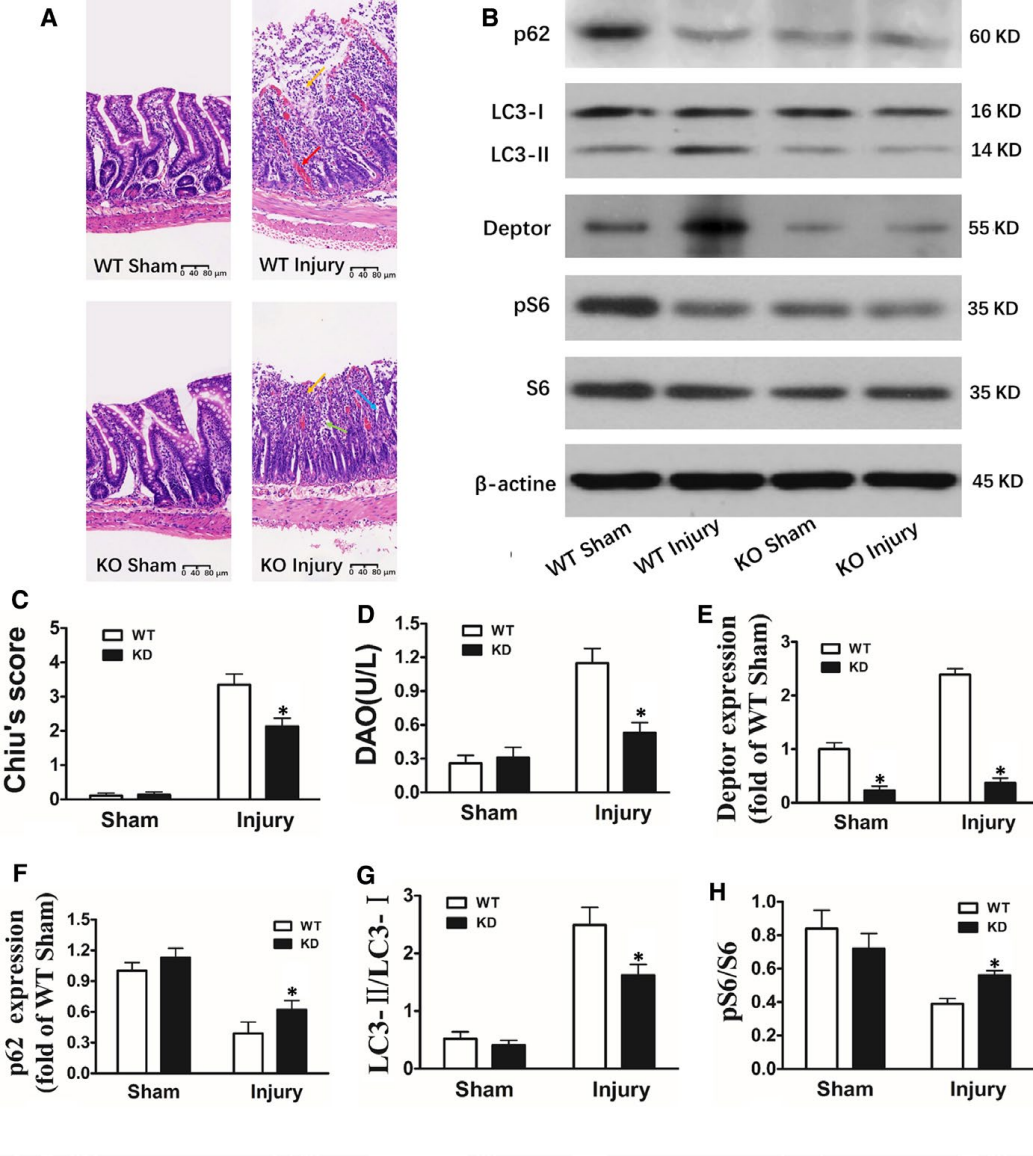
Intestinal I/R injury is reduced, and mTOR activity is enhanced in Deptor KO mice following I/R. Deptor KO mice and WT mice were subjected to intestinal I/R injury. A, Histopathologic changes of the intestinal mucosa (haematoxylin and eosin staining). The intestinal mucosa was intact in the WT Sham and Deptor KO Sham group. Histopathologic changes in WT Injury were consistent with the above description. Development of subepithelial Gruenhagen's space at the apex of the villus, extension of the subepithelial space with moderate lifting of the epithelial layer from the lamina propria and massive epithelial lifting down the sides of villi were observed in the Deptor KO Injury group. B, Representative electrophoresis pattern of p62, LC3‐I, LC3‐II, Deptor, pS6 and S6. C‐F, Changes of Chiu's score, DAO, Deptor, LC3‐II/LC3‐I, p62 and pS6/S6 respectively. G, H, Intestinal I/R injury is reduced, LC3‐II/LC3‐I is enhanced, and pS6/S6 is decreased in Deptor KO mice following I/R. The data were expressed as the mean ± SD (n = 8). *P < .01 compared with the Sham group, ^#^ *P *< .05 compared with the Injury group

To certify the function of Deptor in intestinal I/R damage, Deptor knockout (KO) mice were used. The results showed that Deptor expression in KO mice was only 25% of the wild‐type (WT) mice (Figure 3E). The intestinal injury in Deptor KO mice was ameliorated in comparison with the WT mice following I/R (Figure 3A,C,D). Meanwhile, mTOR activity was enhanced and autophagy was reduced in Deptor KO mice (Figure 3E‐H).

### 3.5 Deptor is a target gene of miR‐182

**FIGURE 4:**
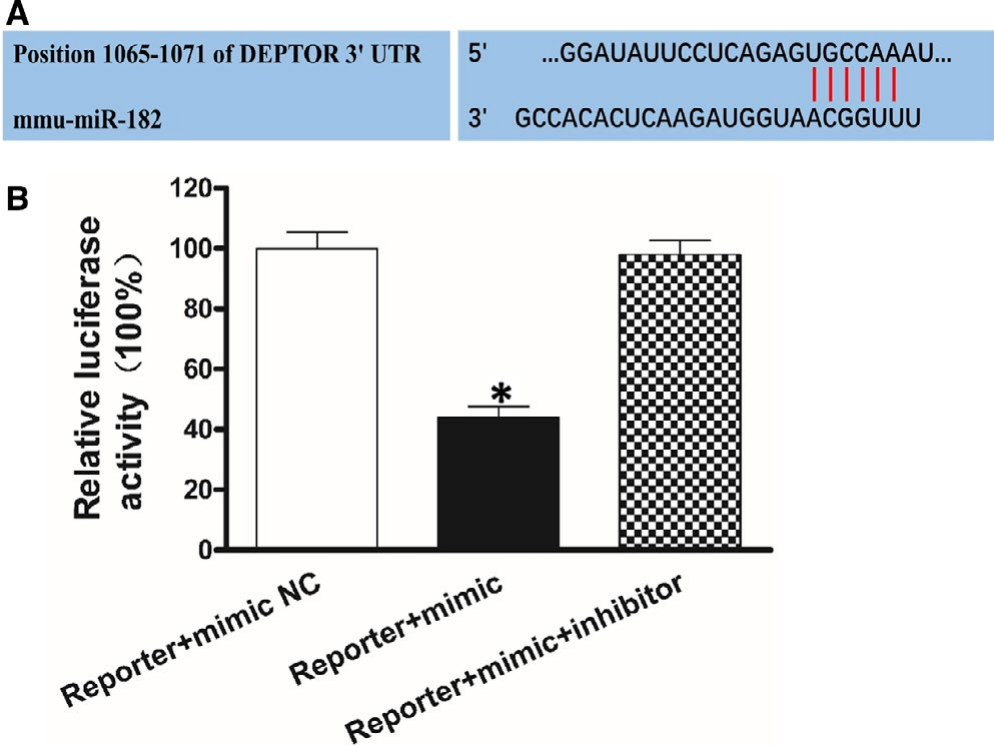
The luciferase reporter assay of miR‐182. miR‐182 mimic, miR‐182 inhibitor and NC mimic were cotransfected with a modified control vector containing the Deptor 3′‐UTR. A, A schematic representation of the interaction between miR‐182 and the 3′‐UTR of Deptor. B, The luciferase assay showed that miR‐182 down‐regulated 44% of the expression of Deptor in the Reporter + mimic miR‐182 group, whereas the miR‐182 inhibitor reversed the effect of miR‐182.The results indicated that miR‐182 acts on the 3′‐UTR. The data are expressed as the mean ± SD (n = 5 independent experiments). *P < .01 compared with the Reporter + mimic NC group

A bioinformatics analysis identified Deptor is a probable target of miR‐182 (Figure 4A). In order to verify the relation between miR‐182 and Deptor, a dual‐luciferase reporter gene assay was tested. In the Deptor 3’UTR WT group, in comparison with the cells transfected negative control, the standard luciferase activity in the simulated transfected cells was obviously lower, but the standard luciferase activity in the inhibitor transfected cells was obviously higher. However, by transfection with either miR‐182 mimics or inhibitors, the activity level of the luciferase construct containing the mutant 3′UTR was not decreased (Figure 4B). The above results showed the miR‐182 expression was inversely related to the expression of Deptor (Figure 1). Collectively, these outcomes indicated that Deptor was a target of miR‐182.

### 3.6 Deptor is indispensable for the effect of mir‐182 on intestine under I/R condition

**FIGURE 5:**
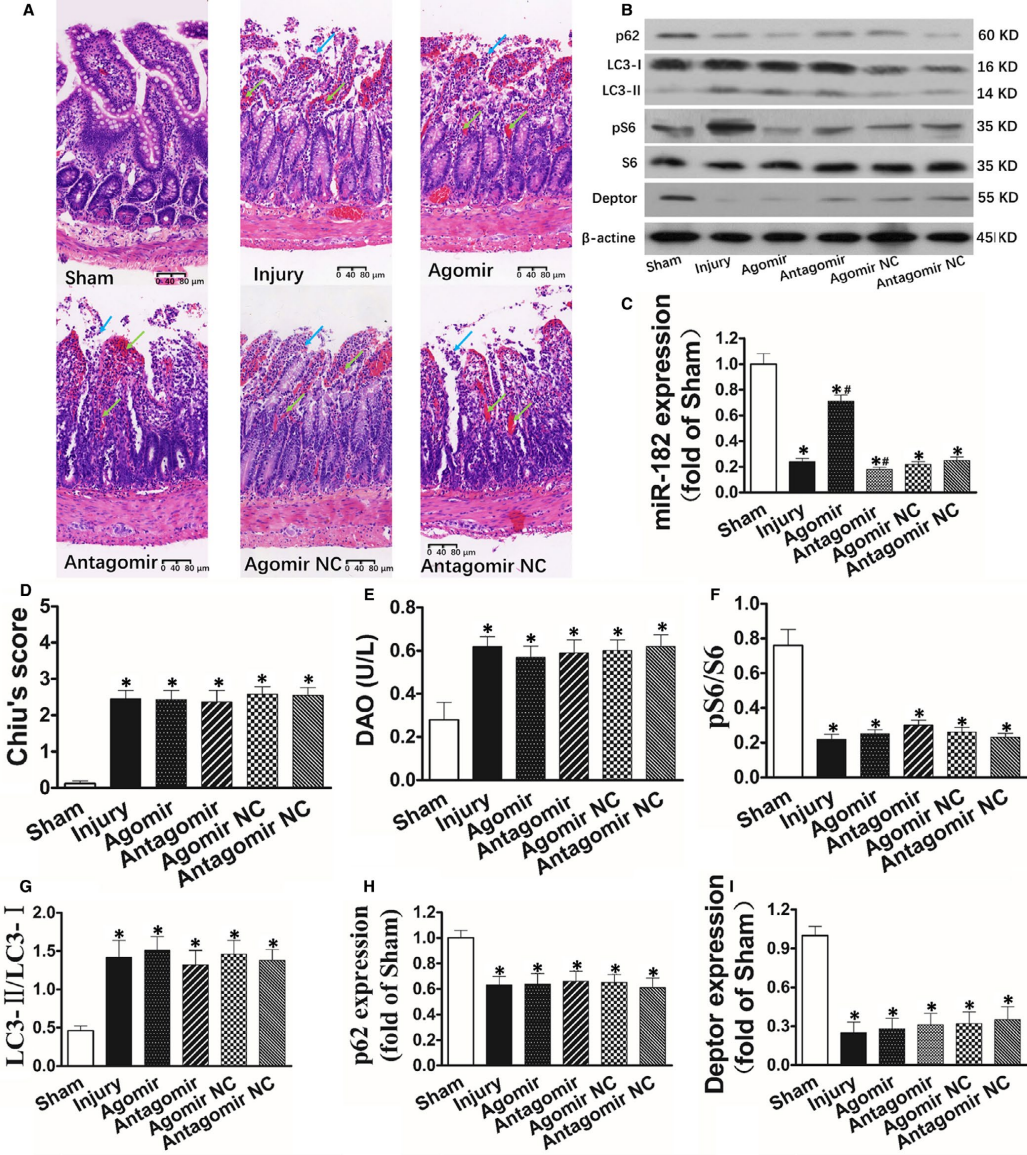
Deptor is indispensable for the effect of miR‐182 on intestine under I/R condition. Deptor KO mice were subjected to intestinal I/R injury. The mice received injections of agomiR‐182, antagomiR‐182 or their NC via the tail vein (40 mg/kg, 100 μL) for three consecutive days. The intestinal I/R injury model was established on the fourth day after injection. A, Histopathologic changes of the intestinal mucosa (haematoxylin and eosin staining). The intestinal mucosa was intact in the sham group, whereas Development of subepithelial Gruenhagen's space at the apex of the villus, often with capillary congestion, and extension of the subepithelial space with moderate lifting of the epithelial layer from the lamina propria were observed in the other groups. B, Representative electrophoresis pattern of p62, LC3‐I, LC3‐II, pS6, S6 and Deptor. C‐I, Changes of miR‐182, Chiu's score, DAO, LC3‐II/LC3‐I, pS6/S6, p62 and Deptor, respectively. The data were expressed as the mean ± SD (n = 8). *P < .01 compared with the Sham group, ^#^ *P *< .05 compared with the Injury group

We next determined whether Deptor/mTOR was responsible for the protective effects induced by miR‐182. As shown in Figure 1, agomir‐182 alleviated intestinal injury under I/R conditions in WT mice. However, up‐regulation of miR‐182 did not confer such protection in Deptor KO mice following I/R (Figure 5). Meanwhile, antagomir‐182 aggravated intestinal injury in WT mice. However, down‐regulation of miR‐182 did not confer such damage in Deptor KO mice after I/R (Figure 5).Collectively, these data revealed that Deptor was required for the regulation of miR‐182 mediated protection from intestinal I/R damage.

## 4 DISCUSSION

Intestinal I/R injury remains challenging in critical patients and is** **regarded as a major cause of multiple organ failure.^1^ However, the precise mechanisms are not wholly understood.^2^ This research presented some significant findings. First, we confirmed that miR‐182 was down‐regulated in intestine following I/R injury and observed that up‐regulation of miR‐182 reduced intestinal injury, autophagy, Deptor expression and enhanced mTOR activity following intestinal I/R. Second, we confirmed that repression of autophagy reduced intestinal injury and inhibition of mTOR aggravated intestinal injury. Third, we observed that intestinal I/R injury and autophagy were decreased, and mTOR activity was enhanced in Deptor KO mice following intestinal I/R. Fourth, mechanistic studies had shown that miR‐182 directly targeted Deptor and Deptor performed an essential role in miR‐182 mediated protection during intestinal I/R. Thus, we concluded that miR‐182 performed protection in intestinal I/R damage and its protective function was mediated by inhibiting autophagy via mTOR by targeting Deptor.

Accumulating evidence suggests miRNAs participate in the procedure of I/R damage. Chen et al^12^ found that miR‐424 reduced kidney injury induced by I/R. Liu et al^13^ found down‐regulation of miRNA‐199a‐5p attenuated cytotoxicity induced by hypoxia/reoxygenation in cardiomyocytes. Our previous study^8^ showed miR‐182 was obviously down‐regulated in the intestine following I/R. miR‐182 exerts important functions and influence in various diseases.^14^ For instance, a large number of studies have showed miR‐182 is expressed in some cancers and is considered as an oncogenic miRNA, which can promote cancer cell survival, proliferation, tumorigenesis, aggressiveness and drug resistance.^15^ It is interesting that miR‐182 played different role in different organs subjected to I/R damage. Yi et al^16^ and Li et al^17^ discovered that miR‐182 aggravated the damage induced by I/R in brain and kidney. By contrast, Jia et al^18^ found that up‐regulation of miR‐182 protected from hypoxia‐induced cardiomyocytes apoptosis. miR‐182 expression can be regulated in some steps, including miRNA biogenesis, transcriptional regulation or indirect regulation by stress or other conditions. Marzec‐Kotarska et al^19^ discovered that amplification of miR‐182 gene was responsible for miR‐182 overexpression in epithelial ovarian cancers. Some long non‐coding RNAs,^20^, ^21^ TGF‐β^22^ and β‐catenin^23^, also can regulate the expression of miR‐182. However, the mechanisms of miR‐182 regulation during I/R are still unknown. Histone methylation and histone deacetylation may be involved in the regulation of miR‐182. Some long non‐coding RNAs and other miRNAs changed by I/R also may be involved in the regulation of miR‐182. DNA methylation at the CpG site in the promoter region may be another mechanism for regulating miR‐182 during I/R. In this study, we observed miR‐182 was obviously decreased in intestine following I/R injury, which was observed in our earlier research.^8^ Further studies showed that intestinal injury was significantly reduced when up‐regulation of miR‐182 while was further exacerbated when down‐regulation of miR‐182. These results indicated up‐regulation of miR‐182 reduced intestinal I/R damage.

Previous evidence showed that autophagy played a protective role by removing damaged and toxic cellular components. However, current studies have identified the function of autophagy in I/R injury remains controversial^3^, ^4^. Some studies have shown inhibition of autophagy reduced myocardial,^24^ renal,^25^ hepatic^26^ and cerebral^27^ I/R injury. However, several studies have shown enhancing autophagy attenuated myocardial,^28^ renal,^29^ hepatic^30^ and cerebral^31^ I/R damage. Till now, there are some researches about autophagy in intestine,^32^ suggesting that autophagy is participated in intestine diseases, but very rare is known about autophagy in intestinal damage caused by I/R. In current study, our results showed autophagy was obviously enhanced in intestine following I/R, which was detected in our previous study.^7^ Moreover, we found that reduction in autophagy reduced intestinal damage following intestinal I/R, which was consistent with Yamoto et al study^33^ but was inconsistent with Wen et al study.^34^ This may be because of several factors, for example the different experimental model of intestinal I/R damage, and the lack of drugs which stimulate or inhibit autophagy specifically. So how pharmacological regulation of intestinal autophagy could ameliorate or aggravate intestinal injury after I/R remains to be proved.

There are many signalling participated in the regulation of autophagy. The most important one in these signalling is mTOR, which acts as an inhibitor of autophagy and a gatekeeper in the process of autophagy.^35^ Increasing evidence suggests that autophagy regulated by mTOR is deeply involved in I/R injury. For example, Xiao et al^36^ discovered that ulinastatin protected cardiomyocytes from I/R injury via regulating autophagy through mTOR activation. Yang et al^37^ found that repression of mTOR suppressed autophagy and ameliorated ischaemic brain damage. Song et al^38^ diacovered miR‐101 mitigated liver I/R damage by suppressing autophagy via activating mTOR pathway. In this present study, we found intestinal mTOR activity was decreased and autophagy was enhanced after I/R injury, and mTOR inhibition with rapamycin treatment prior to ischaemia aggravated intestinal injury, which indicated that mTOR activity inhibition aggravated intestinal I/R injury.

mTOR complex 1 (mTORC1) and mTORC2 are two distinct signalling complexes formed by mTOR. Over the last few years, owing to regulating mTOR naturally and negatively, Deptor has become the focus of research on the occurrence and development of human malignant tumours.^39^ Peterson et al firstly illustrated mTORC1 and mTORC2 were directly bound by Deptor and found that the activity of mTORC1 and mTORC2 was increased when down‐regulation of Deptor while was inhibited when up‐regulation of Deptor.^40^ Obara et al^41^ found Deptor was indispensable for metformin to restrain mTOR activity and cell proliferation. Some researchers have emphasized the key function of Deptor during the maintenance and development of cancer,^42^ obesity,^43^ type 2 diabetes^44^ and neurodegeneration.^45^ Nevertheless, the function of Deptor in I/R damage still remains unknown. In present study, we first investigate the function of Deptor in intestinal damage induced by I/R and found that intestinal damage was ameliorated in Deptor KO mice, which indicated that Deptor inhibition reduced intestinal damage. In addition, mTOR activity was enhanced in Deptor KO mice following intestinal I/R.

As the natural inhibitor of mTOR, participation in apoptosis, proliferation and autophagy are the crucial functions of Deptor. Some results have revealed that Deptor performs a key character in autophagy signalling in answer to some stresses, for example energy depletion, partly via negatively regulating the mTOR activity. Yao et al^46^ found that Deptor up‐regulated sensitivity of pituitary tumour cells to cabergoline, decreased its proliferative functions by promoting autophagy via inhibition of mTOR activity. Tian et al^47^ indicated that induction of autophagy regulated by Deptor/mTOR pathway might be a critical mechanism for glutamine limited the development of colorectal cancer related to olitis. In this present study, mTOR activity was enhanced and autophagy was inhibited in Deptor KO mice following intestinal I/R damage, which indicated that autophagy was inhibited through mTOR activated by Deptor suppression following intestinal I/R injury.

A number of functional results have revealed the expression of Deptor is strictly controlled by many mechanisms. Peterson et al had shown that Deptor and its mRNA expression were negatively regulated by both mTORC1 and mTORC2.^40^ Growth factor‐β,^48^ Che‐1^49^ and Baf60c^50^ have been found to regulate Deptor expression. Recent studies indicated that miRNAs might also regulated expression of Deptor. Chen et al^51^ found miR‐375 stimulated hASCs to osteogenic differentiation by targeting Deptor to repress the activity of AKT. Maity et al^52^ found that miR‐181a inhibited Deptor expression for glomerular gesangial cell cypertrophy and matrix protein expression induced by TGFβ. Through prediction of the target genes, we found that one of the miR‐182 binding sites was predicted within the Deptor mRNA 3′‐UTR, which meant that when increased miR‐182 expression, Deptor expression may decreased. In present research, we discovered that miR‐182 expression negatively regulated Deptor and Deptor mRNA expression. Furthermore, the assay of dual‐luciferase reporter gene further verified that Deptor was a target gene of miR‐182. These outcomes suggested miR‐182 could regulate Deptor directly. The process of miRNA regulating target genes is complex. Study has estimated that the bulk of miRNAs can regulate about 200 mRNAs.^53^ By using prediction algorithms and the assay of dual‐luciferase reporter, the relation between miR‐182 and Deptor in ischaemic intestine had been identified in this study. Further studies showed that agomir‐182 alleviated intestinal injury and antagomir‐182 aggravated intestinal injury under I/R conditions in WT mice. However, up‐regulation or down‐regulation of miR‐182 did not confer such protection or exacerbation in Deptor KO mice. Taken together, these data reveal that Deptor was required for the regulation of miR‐182 mediated prevention of intestinal I/R damage.

There were still some candidate genes for miR‐182 including SOGA1 and Rictor that were closely related to autophagy. SOGA1 had been reported to perform as a protein associated with microtubule, which was related to formation of autophagy.^54^ Rictor is the key component of mTORC2 which plays an vital role in regulating Akt activity. It has been reported the phosphorylation of mTOR triggered by activated Akt regulates cell apoptosis, autophagy and growth.^55^ These genes eventually affect autophagy through complex mechanisms. However, the role of these genes is still further verified by luciferase reports and other experiments in intestinal damage caused by I/R.

In summary, our study showed that up‐regulation of miR‐182 mitigated intestinal I/R injury by inhibited autophagy, and miR‐182 targeted Deptor to repress the mTOR activity during this procedure. These discoveries would reveal new comprehensions into the signalling pathway of intestinal injury induced by I/R and point out miR‐182 is an expected curative target in diseases associated with intestinal damage caused by I/R.

## CONFLICT OF INTEREST

The authors confirm that there are no conflicts of interest.

## AUTHOR CONTRIBUTIONS

Yunsheng Li and Yanhua Luo involved in the study concept and design; analysis and interpretation of data, revising the manuscript for important intellectual content and approval of the final version to be published. Baochuan Li involved in acquisition of data, analysis and interpretation of data, drafting the manuscript and approval of the final version to be published. Lijun Niu involved in acquisition of data, analysis of data, revising the manuscript for important intellectual content and approval of the final version to be published. Jiaxin Liu involved in acquisition of data, interpretation of data, drafting the manuscript and approval of the final version to be published. Xiaoyun Duan involved in acquisition of data, analysis of data, drafting the manuscript and approval of the final version to be published

## Data Availability

Data available on request from the authors. The data that support the findings of this study are available from the corresponding author upon reasonable request.
